# Surfactant‐Associated Protein D Polymorphism and Protection Against COVID‐19 in the Czech Population

**DOI:** 10.1002/iid3.70541

**Published:** 2026-10-06

**Authors:** Jaroslav A. Hubacek, Tom Philipp, Marketa Ondrackova, Vera Adamkova, Ladislav Dusek

**Affiliations:** ^1^ Experimental Medicine Centre Institute for Clinical and Experimental Medicine Prague Czech Republic; ^2^ Third Department of Internal Medicine, 1st Faculty of Medicine Charles University Prague Czech Republic; ^3^ Clinic of Rheumatology and Physiotherapy, 3rd Faculty of Medicine Charles University and Thomayer University Hospital Prague Czech Republic; ^4^ Specialised Outpatient Care Division Institute for Clinical and Experimental Medicine Prague Czech Republic; ^5^ Institute of Health Information and Statistics of the Czech Republic Prague Czech Republic; ^6^ Institute of Biostatistics and Analyses, Faculty of Medicine Masaryk University Brno Czech Republic

Dear Editor,

1

We read with interest the article by Kolsari and colleagues entitled “The Impact of Surfactant Protein‐D Gene Polymorphism on COVID‐19 Clinical Outcomes” [1]. In a sample from the Iranian population they have described a significant association between the *SFTPD* rs721917 TT genotype and T allele and increased risk of hospitalization and severity of symptoms in patients with SARS‐CoV‐2 infection. The TT genotype was more prevalent in ICU and CCU‐admitted patients than the CC genotype (*p* < 0.05) and was linked to higher overall hospitalization rates. However, no significant correlation was found with mortality rates or patients' age.

There is a broad spectrum of clinical manifestations of severe acute respiratory syndrome coronavirus 2 infection (causing COVID‐19), ranging from asymptomatic cases to severe, life‐threatening complications. A significant part of this variation is based on genetic predisposition [2, 3]. Pulmonary surfactant‐associated protein D (SFTPD) plays a role in pulmonary immune defense [4] and is a logical candidate for influencing the course of COVID‐19. To replicate the aforementioned results, we analyzed *SFTPD* polymorphism rs721917 (C/T; Met31/Thr) using PCR‐RFLP in 436 adult patients with confirmed SARS‐CoV‐2 infection (164 asymptomatic and 272 symptomatic) [5, 6] and 467 adult controls (selected as a subset of subjects from the Czech post‐MONICA study [7]). Further methodological details are available from the corresponding author. SARS‐CoV‐2 positivity (in patients) or negativity (in controls) was confirmed by PCR screening and this status was known until December 2024. The SPSS Statistics 27 statistical package was used for statistical analyses.

The distribution of genotypes within our groups has remained within the Hardy–Weinberg equilibrium (*p* = 0.80 for controls and *p* = 0.24 for patients). No significant difference in genotype distribution was observed between the entire group of SARS‐CoV‐2 patients and the control group (*p* = 0.59) (for more details, see Table 1). However, significant differences were observed in genotype frequencies between asymptomatic subjects and controls (*p* = 0.02) as well as between asymptomatic subjects and symptomatic patients (*p* = 0.01). Nonetheless, it should be noted that a higher proportion of heterozygotes within the asymptomatic subjects drives the difference and overdominant effect could not be excluded. It is also of interest to mention that the frequency of the CC genotype increased at a borderline, nonsignificant level (*p* = 0.08 for trend) from the asymptomatic group through the population controls to the symptomatic subjects. Thus, in contrast to a previous study [1] carriers of at least one T allele (in comparison with CC homozygotes) were slightly less likely to be present among the symptomatic COVID‐19 group (OR, 95% CI 0.59 (0.34–1.05), *p* = 0.07). Unfortunately, we do not have any DNA samples from hospitalized or deceased patients with COVID‐19 available.

**Table 1:** Genotype distribution of *SFTPD* polymorphism in COVID‐19 patients and controls.

	SARS‐CoV‐2 positive		COVID‐19 asymptomatic		Controls (population)		COVID‐19 symptomatic		*p*
rs721917	*N*	%	*N*	%	*N*	%	*N*	%	
TT	145	33.3	46	28.0	165	35.3	99	36.4	*0.59
TC	223	51.1	99	60.3	223	47.8	124	45.6	**0.02
CC	68	15.6	19	11.6	79	16.9	49	18.1	***0.84
*p* for HWE	0.24				0.80				****0.01

* SARS‐CoV‐2 positive versus controls.

** Asymptomatic versus controls.

*** Symptomatic versus controls.

**** Asymptomatic versus symptomatic.

The discrepancies between our study and the published study [1] could be explained by the fact that the number of subjects in the original article was relatively low. In fact, the numbers were between 17 and 42 only within all groups (mild, moderate, severe, and critical COVID‐19 course), which makes the study prone to false positive results. In addition, as the genotype frequency within the entire group of subjects is far out of expected Hardy–Weinberg equilibrium (with *p* < 0.0000001), this marked deviation further complicates interpretation and may reflect ascertainment, population structure, or possible genotyping inaccuracies. Last but not least, significant interethnic differences between SARS‐CoV‐2 infection susceptibility are known [8], also this could also be a reason for the discrepancy between our study and the original [1] study.

Finally, it is of major importance to highlight the meta‐analysis [9] of genome‐wide association studies focusing on variants associated with COVID‐19 course. Specifically, the rs721917 variant is listed among the 49 variants associated with critical COVID‐19. Here, the T allele was associated with about a 7% lower risk of critical COVID‐19; thus, rather supporting the results from our case‐control study (where T allele is nominally associated with a 4% decrease). Interestingly, despite the fact that *SFTPD* seems to be a biologically plausible candidate for influencing individual susceptibility to COVID‐19, no further studies on adults, outside GWAS analyses, that focus on this topic were published. In children, no effect of this *SFTPD* polymorphism on COVID‐19 severity was detected [10].

Two studies analyzed concentrations of plasma surfactant protein D (SP‐D) levels and revealed similar results. No difference in plasma concentration of SP‐D was observed between 141 convalescent COVID‐19 patients 10 weeks after infection and 98 uninfected subjects [11] nor were SP‐D levels associated with COVID‐19 severity in 325 children [10].

In conclusion, our findings, along with those of others [9], suggest that carriers of the T allele of the rs721917 polymorphism at the *SFTPD* locus are potentially protected (not at increased risk) against symptomatic or severe forms of COVID‐19. Further studies are required to detect potential ethnic‐specific effects of this variant.

## Author Contributions

**Jaroslav A. Hubacek:** conceptualization, funding acquisition, methodology, investigation, formal analysis, writing – original draft. **Tom Philipp:** supervision, conceptualization, investigation, data curation, writing – review and editing. **Marketa Ondrackova:** DNA analyses, conceptualization, investigation, data curation, writing – review and editing. **Vera Adamkova:** supervision, investigation, data curation, writing – review and editing. **Ladislav Dusek:** supervision, conceptualization, funding acquisition, methodology, investigation, data curation, writing – review and editing.

## Consent

Informed consent was obtained from all subjects involved in the study.

## Ethics Statement

The study was conducted in accordance with the Declaration of Helsinki and approved by the Institutional Ethics Committee of the Institute for Clinical and Experimental Medicine.

## Conflicts of Interest

The authors declare no conflicts of interest.

## Data Availability

For collaboration purposes, data will be made available on request from the corresponding author.
